# COVID-19 prevention and rehabilitation related knowledge and practices among Egyptian post-COVID-19 patients

**DOI:** 10.1371/journal.pone.0292247

**Published:** 2023-10-06

**Authors:** Marwa Rashad Salem, Nelly Hegazy, Shaimaa A. M. Abd El Fatah, Alaaelrahman Essam Mahmoud Shahib, Ahmad Mohamad Hejazi

**Affiliations:** 1 Public Health and Community Medicine Department, Faculty of Medicine, Cairo University, Cairo, Egypt; 2 Public Health, and Community Medicine Department, Faculty of Medicine, Helwan University, Cairo, Egypt; 3 Faculty of Medicine, Cairo University, Cairo, Egypt; 4 Sixth Grade Medical Student, Faculty of Medicine, Cairo University, Cairo, Egypt; Lorestan University of Medical Sciences, ISLAMIC REPUBLIC OF IRAN

## Abstract

**Background:**

Even with the mild form of COVID-19, people need to practice the proper preventive measures to achieve health, safety and control spread of infection. Few studies assessed sound disinfection and rehabilitative knowledge. This study aims to assess the self-reported, knowledge of specific disinfection measures among post-recovery COVID-19 patients and identifying the most requested knowledge items regarding the prevention and post COVID rehabilitation measures.

**Methods:**

This is an exploratory cross-sectional study using an electronically open survey. A pre-tested e-questionnaire was employed for data assembling. The sample size was calculated and a total of 417 people completed the questionnaire. Knowledge score was calculated for preventive and disinfection measures during and after COVID 19 infection. It comprised three sections: socio-demographics, study participants’ knowledge regarding precautions, disinfection, and rehabilitation measures, as well as sources of knowledge about COVID-19.

**Results:**

All participants infected with COVID-19 (82%) reported self-isolation for ten days after confirming the infection, with only 18% required hospitalization. Regarding the information needed by the participants, the highest requests were for the rehabilitation information after COVID-19 and preventive measures. Females under 30 years old and those with a college education or higher were significantly more likely to request rehabilitation information after COVID-19 (P-value 0.05). Nevertheless, males were significantly more interested in information regarding preventive measures; They were over 30 years old and had education below university (P-value 0.05). Participants (above 30 years old) had significantly higher knowledge of preventive and disinfection measures during and after COVID-19 infection (P = 0.030).

**Conclusion:**

After the COVID-19 experience, most participants demonstrated a great desire for rehabilitation information and proper preventive measures. This paves the way for delivering self-management and rehabilitation knowledge and emphasizing the significance of various prevention modalities.

## Introduction

The COVID-19 pandemic had a profound impact on many aspects worldwide. The burden of COVID-19 is contributed to its rapid emergence, the large number of affected populations, the need to intensive care even with the minority with no previous knowledge to its symptoms, signs or complications. Covid- 19 affects different body systems such as the respiratory system, cardiovascular system, and brain, and even it may result in blood clotting. COVID 19 can leave the patients suffering from persistent dysfunction of any of the affected organs [1].

Evidence suggests that 10 to 20% of recovered patients may experience a group of mid-and long-term effects, which are known as Post-COVID -19 condition. This condition can start with the initial illness and persist, or first develop after recovery. Post COVID-19 condition may include ease of fatigability, loss of smell or taste, respiratory symptoms like shortness of breath, persistent cough, and chest pain; cognitive dysfunction like difficulty in concentration, sleep problems, forgetfulness, confusion, and even psychological problems in the form of anxiety and depression [2,3]. Unfortunately, not all patients are fully aware that what they are suffering, is from the residual effects of COVID- 19, and therefore they do not seek medical help. Post COVID-19 could affect the ability of the recovered patients to perform their daily activities and regain their normal lives [3,4].

Therefore, after recovery from COVID-19, people still need to practice the proper preventive measures to maintain health and safety and continue controlling the spread of the disease. However, others may still suffer from the sequel of the affected organs and experience Post Covid 19 condition, even with the mild form of disease, and they will need some rehabilitative measures to restore their original functions [5,6].

COVID-19 knowledge regarding prevention measures in our daily lifestyle has been set by various health organizations such as WHO [7,8]. In this context, WHO has proposed a series of preventive, rehabilitation, and healthy lifestyle guidelines to combat and protect against COVID-19 [9,10]. Few studies assessed sound disinfection and rehabilitative knowledge particularly in developing countries. This study aims to assess the self-reported, current knowledge of specific disinfection measures among post-recovery COVID-19 patients. Besides, identifying the most requested knowledge items regarding the prevention and post COVID rehabilitation measures.

## Materials and methods

### Study design

This is an exploratory cross-sectional study using an electronically open survey performed among the general population during the study duration from March to June 2021. The study was carried out following the Checklist for Reporting Results of Internet E Surveys (CHERRIES) [11].

### Sample size and sampling technique

The researchers used a convenience sampling technique whereby they searched on Facebook for groups with large members. The sample size was calculated using the following formula: n = required sample size, SD**=** 1.96, P = prevalence of the outcome (50%), E = margin of error, 0.05. As there is no similar study focusing on patients’ knowledge after COVID-19. The best assumption (p) was made for the present study to be 50%. Assuming the non-response rate was 10%, a sample of 377 participants was required. However, a total of 417 people completed the questionnaire over this period.

Participants were required to meet the following criteria: i) be Egyptian residents, ii) be adults >18 years old, iii) get infected with COVID-19, and iv) be willing to participate. Incomplete surveys were excluded.

### Data collection tool

A pre-test e-questionnaire (two pages) was used to assemble data. It comprised three sections:

**Section one:** Socio-demographics: age, sex, education, occupation (employed or not), marital status, governorate, and residence.

**Section two:** Knowledge of study participants regarding precautions, disinfection, and rehabilitation measures compiled a total of 30 items in derived from the WHO and the "Do’s and Don’ts" information book; disclosing home treatment, managing activities of daily life, managing stress, anxiety, and self-care [10,12]. Knowledge of some practices during the agony of Covid-19 infection (6 questions), Knowledge about the preventive measures for cleaning and sterilization after recovery (12 questions), knowledge regarding lifestyle changes and rehabilitation after recovery (12 questions).The questions were close-ended with yes, no, and do not know options. The available literature validated the questions used in this section [12,13].

**Section three:** Sources of knowledge about COVID-19 disease comprised various formats, including scientific websites, medical studies, health care providers (HCP), television, Facebook, and WhatsApp. (2 questions).

Two language experts translated the questions into Arabic, then into English by two independent language experts.

Because the COVID-19 crucial situation requires social distancing, the researchers used an online data collection technique. A Google form was created, and participants were encouraged to complete and submit it. The researchers shared the questionnaire link with the groups on Facebook. It is the most popular social media platform in Egypt. To get permission to share this survey, requests were sent to the administrators of these groups. Then, the link of the survey with a post including its objective and the contact information of one of the researchers was shared. This link was accessible for one week. The researchers avoided duplicate entries by preventing users’ access to the survey twice.

A pilot test was conducted with ten participants (not included in the study) to assess the clarity of the questions. The necessary adjustments were made. Four faculty members who are specialists in public health validated the questionnaire’s content, and necessary changes were made. The time needed to fill in the questionnaire was measured. Fifteen minutes was the time frame used as a cutoff point; determined using the pilot test. The questionnaire had an estimated reliability coefficient of (Cronbach’s alpha=0.797).

### Statistical analysis

Statistical Package of Social Science version 24.0 (IBM, SPSS, USA) was used for statistical analysis. Variables were examined for normality. Categorical variables were expressed as proportions and percentages. Quantitative variables were expressed using median and interquartile range (IQR); the Mann-Whitney U and Kruskal-Wallis tests of significance were used for comparison. A p-value of < 0.05 was considered significant.

The score was calculated for 17 items concerned with knowledge and practices of some preventive and disinfection measures during and after COVID 19 infection. The correct knowledge answers were given a score of 1, while wrong answers or answers ending in “I do not know” were given a score of 0. The total raw score (if all answers were correct) was 17. Good knowledge was considered, if participants’ score was above the median score value, while poor knowledge was considered for participants whose score was below the medium score value [14].

### Ethical considerations

The study protocol was approved by the Research Ethical Committee (REC) at Cairo University, Egypt. An electronically signed informed consent was acquired directly from participants before taking the questionnaire and after clarifying the study aims and significance. Confidentiality of data, safe data storage, and privacy rights were considered. All techniques for data collection were handled according to the Helsinki Declaration of biomedical ethics.

## Results

The sample included 417 individuals; of those, 69.5% were females. The majority awarded a university degree or above. Two-thirds of participants were unmarried, half were employed, and the majority lived in the greater urban Cairo region. Three hundred forty-two (82%) reported self-isolation for ten days after confirming the infection, and only 37 (18%) needed hospitalization. Most of the participants recovered after two weeks to one month, and only 13 (3%) were still diseased (Table 1).

**Table 1:** Distribution of the study participants according to their socio-demographic characteristics and course of their disease (n = 417).

Socio-demographic characteristics		Frequency (n = 417)	Percent (%)
**Age in years**	Below 30	266	63.8
	30 and above	151	36.2
**Sex**	Male	127	30.5
	Female	290	69.5
**Educational level**	Below University	69	16.5
	University and above	348	83.5
**Marital status**	Married	156	37.4
	Not married	261	62.6
**Occupation**	Unemployed	167	40.0
	Employed	231	55.4
	Retired	19	4.6
**Residence**	Urban	384	92.1
	Rural	33	7.9
**Residence**	Greater Cairo Region	321	77.0
	Alexandria region	34	8.2
	Delta region	27	6.5
	canal region	24	5.8
	upper Egypt	11	2.6
**Course of COVID 19**			
**Isolation 10 days after infection**	Yes	342	82.0
	No	75	18.0
**Need Hospitalization**	Yes	37	8.9
	No	380	91.1
**Time of recovery from COVID 19**	2weeks- 1 month	274	65.7
	1-2 month	36	8.6
	2-3 months	22	5.3
	more than 3 months	72	17.3
	still diseased	13	3.1

Regarding knowledge about items that could transmit infection and need to be disinfected, most various ages, academic grades, and both sexes significantly reported doorknobs, toilets, basins, and light switches. The use of alcohol at 70% or sodium hypochlorite (1%) to disinfect the home was significantly higher among participants under 30 years old and whose education was above university (Table 2).

**Table 2:** Percent distribution of study participants by their knowledge of some disinfection measures post-COVID-19 infection (n = 417).

Disinfection measures	Age				Sex					Education				Accommodation			
	< 30 Years		≥ 30 Years		Male			Female		Below University		University & above		Urban		Rural	
Items needed to be disinfected																	
	n	%	n	%	n	%	n		%	n	%	n	%	n	%	n	%
Toilet/basin	194	74.3	126	84	92	73.6	228		79.7	58	85.3	262	76.4	295	78	25	75.8
Light switch	187	71.6	80	53.3	63	50.4	204		71.3	39	57.4	228	66.5	244	64.6	23	69.7
Door knob	226	86.6	121	80.7	100	80.0	247		86.4	63	92.6	284	82.8	319	84.4	28	84.8
floor	69	26.4	40	26.7	32	25.6	77		26.9	17	25.0	92	26.8	100	26.5	9	27.3
kitchen surfaces	110	42.1	53	35.3	48	38.4	115		40.2	20	29.4	143	41.7	151	39.9	12	36.4
toys	87	33.3	47	31.3	29	23.2	105		36.7	16	23.5	118	34.4	126	33.3	8	24.2
remote controls	155	59.4	63	42.o	61	48.8	157		54.9	27	39.7	191	55.7	202	53.4	16	48.5
Don’t know	17	6.5	9	6	10	8.0	16		5.6	1	1.5	25	7.3	24	6.3	2	6.1
P value*	< 0.0001*				< 0.0001*					< 0.002*				0.979			
**How to disinfect home/isolation room**																	
Alcohol	214	80.5	94	62.7	88	69.3	220		76.1	44	63.8	264	76.1	281	73.4	27	81.8
Sodium Hypochlorite	147	55.3	94	62.7	64	50.4	177		61.2	43	62.3	198	57.1	223	58.2	18	54.5
Water&	73	27.4	47	31.3	37	29.1	83		28.7	14	20.3	106	30.5	111	29	9	27.3
Home cleaning																	
Water only	0	0.0	3	2.0	1	0.8	2		0.7	0	0.0	3	0.9	3	0.8	0	0.0
Don’t know	19	7.1	3	2.0	7	5.5	15		5.2	8	11.6	14	4.0	21	5.5	1	3.0
P value*	< 0.0001*				0.265					<0.009*				0.854			

* Chi square test Significant if < 0.05

**Fig 1:**
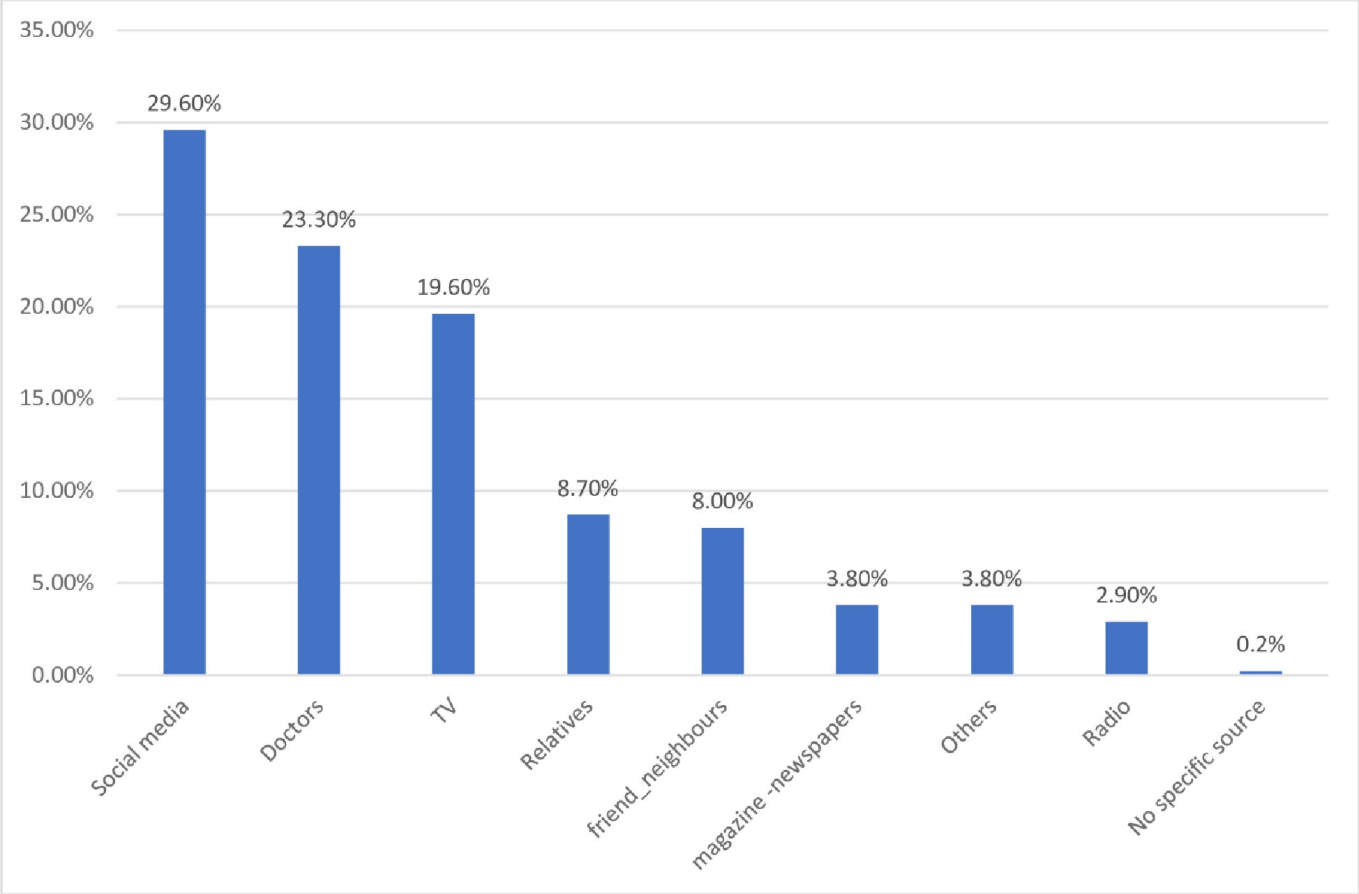
Percent distribution of the enrolled participants by COVID-19 disease source of knowledge (n=417).

Social media, followed by health care professionals (HCPs), were significantly the most common sources of COVID-19 disinfection information (P value< 0.05) (**Fig 1**).

Regarding the information needed by the participants, the highest requests were for rehabilitation information after COVID-19 (28.4%) and the proper preventive measures, other than the disinfection measures, they should continue to practice after recovery (16.9%). Females (60.4%), those under 30 years old (54.9%), and those with a college education or higher (56.9%) were significantly more likely to request rehabilitation information after COVID 19 (P-value <0.05). Males (35.4%) were significantly more interested in preventive measures; they were over 30 years old (43%) and had education below university (46.4%) (P value <0.05) (Table 3).

**Table 3:** Percent distribution of study participants according to their most needed information about COVID-19 and their Post COVID symptoms (n = 417).

Knowledge Items	Age				Sex				Education				Accommodation			
	< 30 Years		≥ 30 Years		Male		Female		Below University		University & above		Urban		Rural	
Information needed by participants																
	N	%	n	%	n	%	n	%	n	%	n	%	n	%	n	%
Preventive measures	70	26.5	65	43.0	45	35.4	90	31.3	32	46.4	103	29.8	34	9%	3	9.1
Mode of transmission	58	22.0	64	42.4	36	28.3	86	29.9	32	46.4	90	26.0	126	33.0	9	27.3
Proper _ isolation	72	27.3	49	32.5	44	34.6	77	26.7	30	43.5	91	26.3	113	29.6	9	27.3
The Proper disinfection	80	30.3	36	23.8	31	24.4	85	29.5	17	24.6	99	28.6	110	28.8	11	33.3
Rehabilitation	145	54.9	82	54.3	53	41.7	174	60.4	30	43.5	197	56.9	104	27.2	12	36.4
Others	7	2.7	5	3.3	6	4.7	6	2.1	1	1.4	11	3.2	206	53.9	21	63.6
No information needed	55	20.8	12	7.9	24	18.9	43	14.9	5	7.2	62	17.9	9	2.4	3	9.1
P value*	< 0.0001*				0.005*				< 0.0001*				0.221			
**Post COVID symptoms**																
No symptoms	108	40.6	43	28.7	66	52.0	85	29.4	26	37.7	125	36	140	36.6	11	33.3
Dyspnea	53	19.9	44	29.3	12	9.4	85	29.4	5	7.2	92	26.5	94	24.5	3	9.1
Change voice	13	4.9	9	6.0	4	3.1	18	6.2	3	4.3	19	5.5	20	5.2	2	6.1
Easy fatigability	67	25.2	56	37.3	26	20.5	97	33.6	19	27.5	104	30	111	29.0	12	36.4
Difficult eating	15	5.6	11	7.3	9	7.1	17	5.9	6	8.7	20	5.8	24	6.3	2	6.1
Memory problems	55	20.7	36	24.0	15	11.8	76	26.3	10	14.5	81	23.3	87	22.7	4	12.1
Difficult back to work	34	12.8	29	19.3	10	7.9	53	18.3	9	13.0	54	15.6	61	15.9	2	6.1
Anxiety	57	21.4	53	35.3	23	18.1	87	30.1	16	23.2	94	27.1	106	27.7	4	12.1
Others	38	14.3	12	8.0	14	11.0	36	12.5	12	17.4	38	11.0	44	11.5	6	18.2
P value*	< 0.0001*				< 0.0001*				0.027*				0.109			

* Chi square test Significant if < 0.05

**Fig 2:**
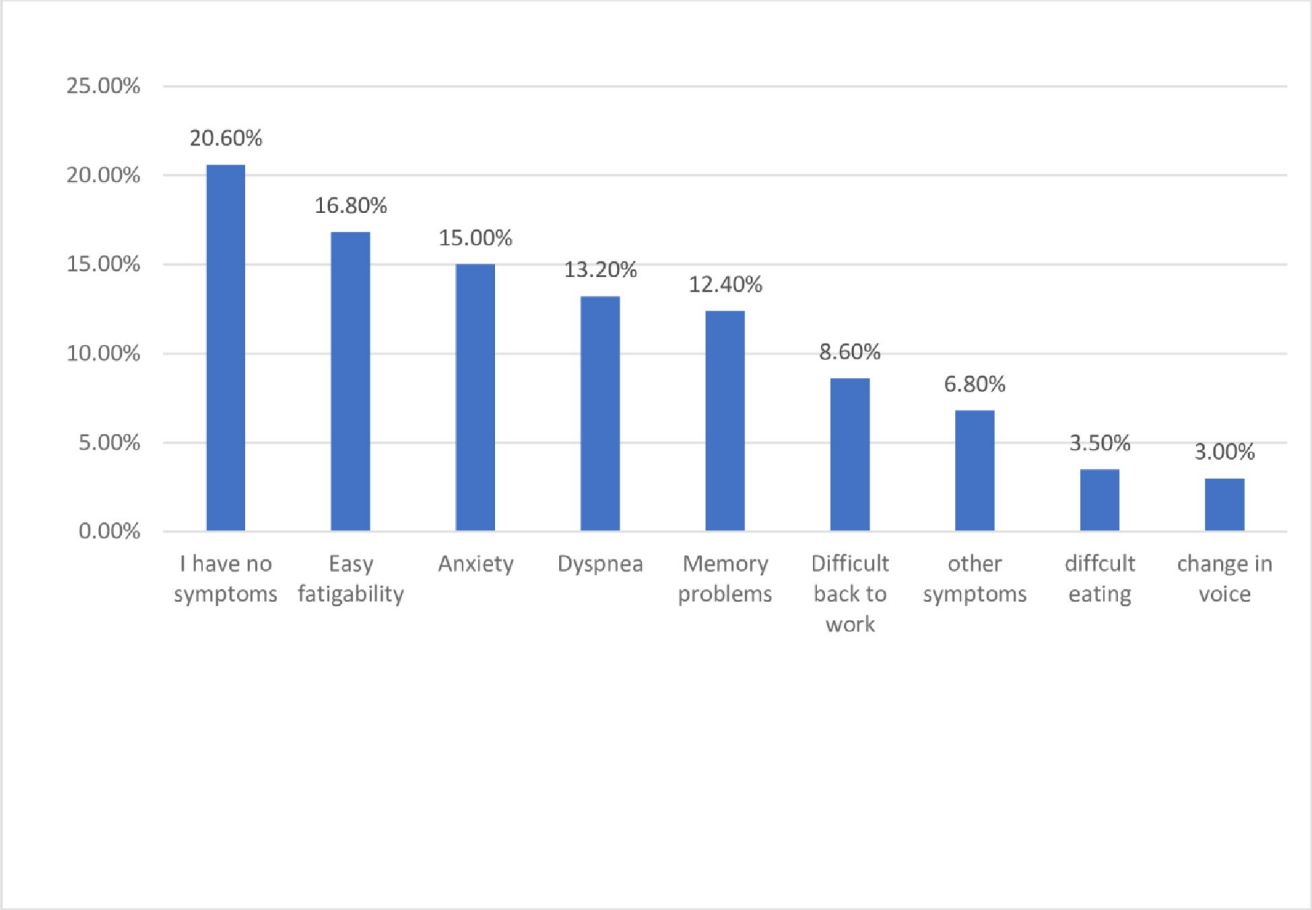
Percent distribution of the enrolled participants by their experienced Post COVID 19 symptoms (n=417).

Regarding the post-COVID symptoms, easy fatigability and anxiety were the most common ones among participants (16.8% and 15%, respectively) (**Fig 2**).

They were significantly high among females (33.6% and 30%), aged 30 years old and above (37.3% and 35.3), having a university degree or above (30% and 27%), and married (35% and 34.2%) (Table 3).

Table 4 demonstrates knowledge scores of some preventive and disinfection measures during and after COVID 19 infection (median = 8 and IQR = 6-10). It was significantly higher among participants of 30 years old and above (P = 0.030); there was no significant association with other sociodemographic characteristics.

**Table 4:** Relationship between COVID 19 knowledge score and sociodemographic characteristics among the enrolled participants (n = 417).

Socio-demographic variables	Knowledge score Median(IQR)	P-value
Age*		
< 30	8(6-10)	0.030*
≥ 30	8(7,10)	
Sex*		
Male	8(6,10)	**0.618**
Female	8(6,10)	
Education*		
Below University	9(7,10)	**0.314**
University and above	8(6,10)	
Occupation*		
Employed	8(6,10)	**0.096**
Not employed	8(6,9)	
Retired	10(8,11)	
Marital status*		
Married	8(7,10)	**0.331**
Unmarried	8(6,10)	
Residence*		
Urban	8(6,10)	**0.072**
Rural	7(6,9)	
Governorate’s region**		
Greater Cairo Region	8(6,10)	**0 .557**
Alexandria region	8(6,9)	
Delta region	8(6,10)	
**C**anal region	8(7,10)	
**U**pper Egypt	8(6,10)	

* Mann-Whitney test significant if < 0.05

**Kruskawallis test significant if < 0.05

## Discussion

The current study investigated the self-reported, post-recovery COVID-19 patients’ knowledge of specific disinfection measures. Also, it inquired about the knowledge items regarding the prevention of infection spread and post COVID rehabilitation measures.

Self-isolation after confirming the infection reflected their realization of two major facts: Firstly, being a citizen in a developing country means being prepared for any circumstances, very high demand of frontline doctors, nurses, and various precautions with the consequent huge shortage in various healthcare facilities [5]. Secondly, home isolation of mild illnesses cases has been necessitated as a prevention choice to lessen the overburden on COVID-19 hospitals [15,16]. Good disinfection knowledge presented in the current study resulted from the unprecedented worldwide situation imposed by the pandemic, which drove people to rethink about their choices and behavior. COVID-19 has made people enthusiastic regarding the coronavirus scientific knowledge (such as its persistence on surfaces as plastic, metal, or glass for up to 9 days, efficient inactivation by various disinfection techniques with 62–71% ethanol, 0.5% hydrogen peroxide, or 0.1% sodium hypochlorite within 1 min). More people have required detailed information from experts who proposed a vast roam of effective and accessible disinfectants that could be utilized to decontaminate surfaces for corona viruses [17,18].

The current study agrees with studies that considered various prevention modalities the most effective strategy to combat COVID-19 in the current conditions. Proper spreading of preventive behaviors represented the primary precautionary modality adopted to control COVID-19 spread and transmission [7,19,20]. More knowledge regarding preventive measures by the current study participants represented their sincere intentions of more proficient behavioral alterations in their daily lifestyle. In developing countries, raising awareness of the disease and its dangers was appropriate to stop its spread and transmission [21].

The good knowledge scores demonstrated in the current study are consistent with studies that demonstrated that the sharing of the COVID-19 information was a superior preventive action to cease its propagation. Various information (such as regions affected in each country, number of infected cases, casualties) were always available in comprehensive, quick, and renewed reports on easily accessible online platforms [7,22,23].

The present study agrees with studies showing that social media activism spread the containment methods and primary guidelines to deal with the COVID-19 pandemic [5,24,25]. Using online platforms has become a more distinct and influential tool to communicate social discussions and comprehend this historical global crisis because people are moving out of physical public places. However, a previous study showed that bots’ automated accounts might circulate certain information on social media platforms to magnify certain discourses and chats at the expense of COVID-19, including loathing hashtags beside COVID-19 content [26,27].

Post COVID-19 condition is now considered of public health importance; it can affect the mildly affected patients with SARS-CoV-2, who represent about 81% in the outpatient settings [28].

Easy fatigability, anxiety and shortness of breath were demonstrated among the participants to be the most experienced post-COVID symptoms. these finding were in accordance with similar studies which tracked the prevalence of the long-term health effects of COVID 19 among non-hospitalized patients, weeks and months after the initial infection [28,29]. Female participants reported higher frequencies for Post COVID 19 symptoms than male participants, possibly due to the difference in immune responses and to traumatic events between the sexes [30,31]. Furthermore, the higher ages have experienced more long-term consequences than the younger ones, which may be due to the existence of underlying health conditions prior to COVID-19, more with older age, increase their probability of developing Post COVID 19 Syndrome [32].

The eagerness of the post-COVID participants for information on preventive measures as per studies that illustrated that individuals’ fear and perceived susceptibility to infection were the main predictors for requesting COVID-19 preventive behaviors [20,33,34]. Nevertheless, another study demonstrated that perceiving the severity of infection was the main drive to know more about preventive measures [35].

Furthermore, the needs of participants above 30 years agree with studies that revealed the increasing sense of being from the high-risk categories among adults. Thus, they executed those steps once they realized that the recommended COVID-19 prevention steps were valid and applicable [36,37]. Besides, the demands of the less-educated adults in our study agree with studies that showed that patients with low literacy had a lower perception of COVID-19 prevention methods than highly educated patients. As a result, they better understood the disease and recommended prevention methods. Health knowledge is a critical and determining factor in implementing COVID-19 preventive behavior [20,38]. This was almost identical to previous studies conducted in Egypt, India, Ethiopia, and Australia [20,39–41].

Male patients had insufficient knowledge regarding COVID-19 preventive manners compared to females; as a result, they sought more prevention information. Multiple study findings supported this; for example, in Hong Kong and Chicago, female patients maintained social distance more than males [42,43].

In the current study, rehabilitation information after COVID-19 recovery came first and was significantly required by young, educated, and female participants. This followed a previous Egyptian study investigating various grades of activity confinement post-COVID-19 recoveries based on post-COVID-19 Functional Status. Young participants (30 years old) and female participants suffered more than males [44]. This excessive suffering by these age and gender categories explained why they sought rehabilitation information. A multidisciplinary rehabilitative program should be tailored for each patient based upon features such as age, COVID-19 illness grade, and chronic diseases. Rehabilitation management targets accelerated recovery after treatment, promoting the quality of life by ameliorating emotional and cognitive domains, improving respiratory function, and minimizing the rate of long-term disability and complications [45–47]. The psychological support of family members and caregivers should also be considered [48].

## Conclusion

The current study revealed that most participants reported self-isolation for ten days after confirming the infection. They gave the higher requests for rehabilitation information after COVID-19 and proper preventive measures more than the disinfection measures, they should continue to practice after recovery. Easy fatigability and anxiety were the most common post-COVID-19 symptoms among participants. Social media, followed by HCP, were the most common sources of COVID-19 information. Such study findings highlights the importance of communicating self-management knowledge and behaviors through distant e-learning or telehealth to achieve comprehensive recovery post-COVID-19. Sound prevention knowledge and post-COVID-19 rehabilitation behaviors represent the best examples for accurate information that must be circulated online.

### Limitations

The results should be seen in the context of the online nature of the survey, which is confined to citizens active on social media. Therefore, large scale prospective research in various Egyptian governorates is required to investigate further citizens’ knowledge regarding COVID-19, Post COVID- 19 Syndrome, and the need for rehabilitation.

## Supporting information

S1 File (DOCX)

S1 Data (XLSX)
