# Supplementary material for: COVID-19 prevention and rehabilitation related knowledge and practices among Egyptian post-COVID-19 patients
Source: PLoS One. 2023 Oct 6;18(10):e0292247. doi: 10.1371/journal.pone.0292247 (PMC10558073; doi:10.1371/journal.pone.0292247)
Supplement: S1 File — (DOCX) [file pone.0292247.s001.docx]

**Questionnaire to assess the prevention and rehabilitation Related Knowledge and Practices among Post-COVID-19 Patients**

Dear participant, this questionnaire is part of a research study to assess the current knowledge of specific disinfection measures and identify the most requested items regarding the prevention and rehabilitation measures among post-recovery COVID-19 patients. This study will help us to identify the knowledge gaps to prepare the required educational messages to satisfy the needs.

If you agree to continue, you consent to the use of your data in this research study. We assure you that your identity is completely anonymous and that your data is anonymous and will only be accessed by the study researchers. By clicking Continue below, you agree to participate in the study.

| N. | Questions | Answers |
| --- | --- | --- |
| 1. | Sociodemographic Data | |
| 1.1 | Age in Years | 1- 18-29 years  2- 30-39 years  3- 40-49 years  4-50-59 years  5-60-69 years  6- 70 years and more |
| 1.2 | Gender | 1. Male 2. Female |
| 1.3 | Education | 1. Illiterate 2. Read and write 3. Primary 4. Preparatory 5. Secondary/diploma 6. University graduates 7. Postgraduate studies |
| 1.4 | Working status | 1. Not Working 2. working 3. Retired |
| 1.5 | Current marital status | 1. Married 2. Divorced 3. Widow 4. Single |
| 1.6 | Residence | Name of Governorates..............(list of Governorates |
| 1.7 | Living In Urban/Rural | 1. Urban 2. Rural |

| N. | Questions | Answers |
| --- | --- | --- |
| 2. | Knowledge and some practices regarding the infection with the new Covid-19 virus (Covid-19) | |
| 2.1 | Have you had coronavirus (COVID-19) (symptoms fever - cough - muscle aches - malaise - change/loss of smell or taste) or have you confirmed that you have a virus with a PCR test even without symptoms? | 1. Yes 2. No |
| 2.2 | Did you isolate yourself when infected in a separate room at home for at least 10 days from the onset of illness? | 1. Yes 2. No |
| 2.3 | Have you been admitted to the hospital? | 1. Yes 2. No |
| 2.4 | When did you recover from covid 19 disease?  (Recovery is within 10 days from the onset of symptoms, and at least the last 24 hours, there is no fever without using antipyretic, with the improvement of the rest of the symptoms, or two negative swabs, 24 hours apart) | 1. From two weeks to one month 2. From one to two months 3. From two to three months 4. More than 3 months |
| 2.5 | Did you share your belongings such as bed linen, cell phones, TV remote control, utensils, and glasses with others, during 14 days of isolation? | 1. Yes 2. No 3. I don’t know |
| 2.6 | Did you use public transportation when going to the clinic for an examination or after leaving the hospital to go home? | 1. Yes 2. No 3. I don’t know |

| N. | Questions | Answers |
| --- | --- | --- |
| 3. | Knowledge about the preventive measures for cleaning and sterilization after recovery from Covid 19 disease | |
| 3.1 | Do you know how to disinfect your isolation room or home after recovery? | 1. Yes 2. No 3. I don’t know |
| 3.2 | Is it necessary to wear a mask when disinfecting your isolation room or home after recovery? | 1. Yes 2. No 3. I don’t know |
| 3.3 | In your opinion, cleaning only without disinfection will be enough for the isolation room or the house if they are left 24 hours | 1. Yes 2. No 3. I don’t know |
| 3.4 | Can you pick out the most frequently used items that can transmit infection and need to be disinfected? (You can choose more than one item) | 1. The sanitary place in the bathroom (toilet seat - sink). 2. light switches 3. door handles 4. floors 5. Kitchen surface 6. Children's games 7. Remote control unit for electrical appliances 8. I don't know |
| 3.5 | In your opinion, how can you disinfect the house after recovery before sharing it with others? (You can choose more than one item) | 1. Disinfectants containing 70% alcohol 2. Water and any household cleaner 3. 1% sodium hypochlorite 4. Only water is enough 5. I don't know |
| 3.6 | It is not safe to wash contaminated clothes from a sick person with the clothes of other healthy people. | 1. Yes 2. No 3. I don’t know |
| 3.7 | Warm water should be used to wash the contaminated person's belongings and dry them well by sunlight exposure or using an electric iron. | 1. Yes 2. No 3. I don’t know |
| 3.8 | If you have a sick person at home, use a designated bag-lined trash can and use gloves when removing trash bags. | 1. Yes 2. No 3. I don’t know |
| 3.9 | Do you make sure to wash your hands properly for 20 seconds with soap and water or an alcohol-based hand sanitizer containing at least 70% alcohol regularly at home or after returning from outside after recovering? | 1. Yes 2. No 3. I don’t know |
| 3.10 | Do you continue to wear a mask when you get out of home after recovery? | 1. Yes 2. No 3. I don’t know |
| 3.11 | What is the source of your information about the emerging coronavirus and methods of prevention (you can choose more than one source) | 1. TV 2. Radio 3. Newspapers/magazines 4. The doctor 5. Relatives 6. Friends/neighbors 7. Social media 8. others 9. I do not know |
| 3.12 | In your opinion, what are the most credible sources of information related to the emerging coronavirus? (You can choose more than one source) | 1. Ministry of Health and Population 2. The doctor 3. Internet and social media 4. Foreign media 5. Relatives and friends 6. Clergy (in the mosque or church) 7. I don't know |
| 3.13 | Do you feel you need to know more information regarding the novel coronavirus? (If the answer is no, go to the fourth section of the questionnaire) | 1. Yes 2. No |
| 3.14 | If yes, what do you want to know? (You can choose more than one answer) | 1. The effective preventive measures to prevent infection 2. Methods of transmission of the Coronavirus 3. Proper isolation methods and ways to protect others 4. Ways to disinfect isolation rooms and the house after recovery 5. other |

| N. | Questions | Answers |
| --- | --- | --- |
| 4. | knowledge regarding Lifestyle changes and rehabilitation after recovery | |
| 4.1 | Have you had any of the post-COVID-19 symptoms? (You can choose more than one) | 1. No 2. Difficulty Breathing 3. A change in the voice 4. Ease fatigability and general weakness, tremors, and imbalance 5. Some difficulty in swallowing food and drink 6. Difficult in concentration, memory problems, and brain fog 7. Difficulty returning to education/work / daily activities 8. Stress, anxiety, and depression |
| 4.2 | Should you, after recovery, break your usual routine activities and duties into phases to accomplish? | 1. Yes 2. No |
| 4.3 | Do you ask for help from others to perform your daily routine activities? | 1. Yes 2. No |
| 4.4 | Did your doctor recommend certain sitting positions to improve your breathing? | 1. Yes 2. No 3. I don’t suffer from breathing difficulty |
| 4.5 | Have you started any physical activity (such as walking, running, etc.) to improve your fitness after recovery? | 1. Yes 2. No 3. I don't know the proper fitness exercises after recovery |
| 4.6 | Have you started doing muscle-resistance exercises that help you improve the strength of your muscles after illness? | 1. Yes 2. No 3. I don't know the proper muscle strengthening exercises |
| 4.7 | Have you become interested in eating a balanced diet daily, and home-cooked foods with more fresh fruits and vegetables after recovery? | 1. Yes 2. No 3. I don’t know |
| 4.8 | Are you keen to hydrate your body by drinking (6-8 glasses) of water daily? | 1. Yes 2. No 3. I don’t know |
| 4.9 | Have you become keener to get enough sleep (6-8) hours per day? | 1. Yes 2. No |
| 4.10 | Could you stay in social contact with your family and friends over the phone and the internet to maintain social distancing after recovery? | 1. Yes 2. No 3. I don’t know |
| 4.11 | Do you intend to get the vaccine against the emerging coronavirus after recovery? | 1. yes 2. No, because I am now immune to the new coronavirus and will not be exposed to the infection again 3. No, I don't trust the vaccine 4. No, because of the fear of side effects 5. No, because I believe that preventive measures alone are sufficient to protect me 6. No, I do not have enough information about this vaccine |
| 4.12 | If you are a smoker, have you decided to quit smoking after recovery? (If the answer is no, skip this question, and the questionnaire has ended) | 1. Yes 2. No 3. I don’t know |
